# Resting state EEG in young children with Tuberous Sclerosis Complex

**DOI:** 10.21203/rs.3.rs-4543112/v1

**Published:** 2024-06-27

**Authors:** Caitlin C Clements, Anne-Michelle Engelstad, Carol L. Wilkinson, Carly Hyde, Megan Hartney, Alexandra Simmons, Helen Tager-Flusberg, Shafali Jeste, Charles A Nelson

**Affiliations:** University of Notre Dame; Harvard University; Boston Children’s Hospital; UCLA Health; Boston Children’s Hospital; Boston Children’s Hospital; Boston University; Children’s Hospital of Los Angeles; Boston Children’s Hospital

**Keywords:** Tuberous Sclerosis Complex, seizures, epilepsy, EEG, GABA agonists, biomarker

## Abstract

**Background:**

Tuberous Sclerosis Complex (TSC) manifests behaviorally with features of autism, epilepsy, and intellectual disability. Resting state electroencephalography (EEG) offers a window into neural oscillatory activity and may serve as an intermediate biomarker between gene expression and behavioral manifestations. Such a biomarker could be useful in clinical trials as an endpoint or predictor of treatment response. However, seizures and antiepileptic medications also affect resting neural oscillatory activity and could undermine the utility of resting state EEG features as biomarkers in neurodevelopmental disorders such as TSC.

**Methods:**

This paper compares resting state EEG features in a cross-sectional cohort of young children with TSC (n=49, ages 12–37 months) to 49 age- and sex-matched typically developing controls. Within children with TSC, associations were examined between resting state EEG features, seizure severity composite score, and use of GABA agonists.

**Results:**

Compared to matched typically developing controls, children with TSC showed significantly greater alpha and beta power in permutation cluster analyses iterated across a broad frequency range (2–50Hz). Children with TSC also showed significantly greater aperiodic offset after power spectra were parameterized using SpecParam into aperiodic and periodic components. Within children with TSC, greater seizure severity was significantly related to increased periodic peak beta power. Use of GABA agonists was also independently and significantly associated with increased periodic peak beta power; the interaction between seizure severity and GABA agonist use had no significant effect on peak beta power.

**Conclusions:**

The elevated peak beta power observed in children with TSC compared to matched typically developing controls may be driven by both seizures and GABA agonist use. It is recommended to collect seizure and mediation data alongside EEG data for clinical trials. These results highlight the challenge of using resting state EEG features as biomarkers in trials with neurodevelopmental disabilities when epilepsy and anti-epileptic medication are common.

## BACKGROUND

Electroencephalography (EEG) offers a low-cost, noninvasive neuroimaging method that is feasible in people of all ages and abilities. As such, EEG holds promise as a potential tool to examine brain-based biomarkers for early diagnosis or prediction of treatment response for neurodevelopmental disorders. EEG-based biomarkers could be particularly useful as an endpoint in clinical trials in rare genetic syndromes.

Many children with neurodevelopmental disorders have medical co-morbidities such as epilepsy. Many experience seizures (1) and may be prescribed EEG-altering antiepileptic medications, threatening the use of reliable resting EEG biomarkers as outcome measures in clinical trials of neurodevelopmental disabilities. There is a high prevalence of seizures in neurodevelopmental disabilities with known genetic origins such as Rett Syndrome (60–80% (2)), Fragile X Syndrome (20% (1)), 22q11.2 Deletion Syndrome (11% (3)), CDKL5 Deficiency Disorder (nearly 100% (1), and others, as well as in neurodevelopmental disabilities of unspecified etiology.

### Tuberous Sclerosis Complex

One neurodevelopmental disorder in which seizures are particularly frequent is Tuberous Sclerosis Complex (TSC). Tuberous Sclerosis Complex (TSC) is a rare autosomal dominant disorder that results from mutations in the *TSC1* or *TSC2* genes. TSC has a prevalence of approximately 1 in 7,000 births and is usually detected in utero or the first year of life (4). The inactivation of *TSC1/TSC2* leads to overactivation of the mammalian target of rapamycin (mTOR) pathway, which in turn causes unchecked cell growth and proliferation in some regions of the body, particularly the heart, kidneys, skin, and brain. In the brain, individuals with TSC show hamartomas, including cortical tubers, that impact neuronal function and connectivity (5). Most children with TSC have epilepsy (70–80% (6)) and co-occurring autism (between 46–66% (7)).

Previous EEG research on TSC has identified less maturity and connectivity in the early EEG power spectrum (8,9), and some of these differences have predicted later cognitive development and autistic features (8,10). Studies of older children, adolescents, and adults have reported alterations in connectivity (11–13) and task-based differences (14,15). In a younger cross-sectional sample of 10 toddlers with TSC and 12 typically developing children ages 18–30 months, Stamoulis et al. (2015) noted possible delayed maturation as indexed by a developmental shift of the dominant high-frequency spectral content that occurred later in children with TSC compared to typically developing controls. Children with TSC maintained higher frequencies at older ages, with non-random EEG components present in the high gamma (>50 Hz) and ripple (>80 Hz) frequencies (9). De Ridder et al (2020) also reported early dysmaturity in a clinical sample from the EPISTOP study using a different proxy of maturity. Using neonatal EEGs and 24-month developmental assessment data in 64 children with TSC, De Ridder and colleagues reported that more dysmaturity (indexed by power, range EEG, entropy, and Hurst exponent) predicted more autism traits, as well as lower cognitive, language, and motor developmental scores at 24 months (8). In a similar vein, Dickinson et al. (2019) examined neural network development via features of alpha band oscillations (alpha power, peak alpha frequency, and alpha phase coherence) in a longitudinal sample from 12 to 36 months of 35 toddlers with TSC and 20 typically developing toddlers. Toddlers with TSC showed reduced interhemispheric alpha phase coherence at 12 and 24 months, and the difference was more pronounced at 24 months in TSC toddlers later diagnosed with autism. Peak alpha frequency at 24 months predicted 36 month nonverbal and verbal cognition in both TSC and typically developing children (10).

### This study

None of these prior reports accounted for potential effects of seizure severity or medication on the power spectrum. Seizures affect the power spectrum in a number of ways depending on seizure type (16,17). GABA agonists (*e.g*., vigabatrin) are commonly prescribed to control seizures and increase inhibitory activity as reflected by an increase in beta power (12–30Hz) (18). Most children with TSC have epilepsy and are prescribed GABA agonists and other medications to control seizures. Families increasingly report that their greatest concerns are the TSC-associated neuropsychiatric disorders (TAND) that many individuals with TSC exhibit, such as autism and intellectual disability. Many children with TSC will receive early intervention or other services to support development generally and social communication skills in particular, even among those who do not meet diagnostic criteria for autism. This paper reports on data collected at the first timepoint of JETS (JASPER Early Intervention for Tuberous Sclerosis Complex Study), a randomized controlled trial (RCT) of a behavioral intervention (JASPER: Joint Attention, Symbolic Play, Engagement & Regulation (19)) targeting social communication skills in toddlers with TSC (NCT03422367). A primary aim of the larger RCT is to use EEG data to inform which children with TSC might respond best to a behavioral intervention like JASPER (i.e., a predictive biomarker) and/or to understand neural mechanisms underlying treatment response. Seizures and GABAergic medication use (i.e., antiepileptics and anxiolytics such as benzodiazepines) pose large – but not insurmountable – challenges to using resting EEG spectral power as a biomarker in RCTs for neurodevelopmental disorders. Before resting EEG spectral power features can be employed as biomarkers in a clinical trial, the nature of resting state EEG power must be understood, along with the effects of seizures and medications on the TSC resting state power spectrum.

### Objectives

The aims of the current study are 1) to compare resting state EEG power in toddlers with TSC to age- and sex- matched typically developing children; and 2) to quantify the effects of seizures and medications on the TSC resting state power spectrum. We hypothesize that GABAergic medications will be associated with increased inhibitory activity in children with TSC as reflected by increased beta power.

## METHODS

### Participants

Young children with TSC were recruited into a multisite randomized control trial of a behavioral intervention to target social communication skills (JASPER: Joint Attention, Symbolic Play, Engagement & Regulation (19)) between 2017 and 2023. Children traveled to one of the two participating sites (Boston Children’s Hospital or UCLA Health) for a two-day baseline in-person assessment that included clinical characterization and EEG data collection. At baseline, participants were randomized to receive the 3-month remote-delivery intervention either immediately, or after a 3-month waiting period and repeat assessment. This manuscript reports baseline data only, as intervention is ongoing for the RCT.

Children between ages 12 and 56 months with a clinical diagnosis of TSC diagnosis were eligible for the RCT. Two individuals with TSC were excluded from this analysis to facilitate matching with a typically developing control cohort, so the final age range of children included in this cross-sectional analysis was 12–37 months (M = 22.2(7.4) months; Table 1). Consistent with the literature, 40.8% of TSC participants were reported to have experienced at least one seizure in the last month, 67.4% currently or previously experienced infantile spasms, and 96% were taking at least one medication (M=2.4(1.4) medication). MRI and tuber location were not available.

A comparison cohort of typically developing (TD) children was drawn from the Infant Screening Project 2 (ISP2), a prospective study of autistic and typical development that was conducted at Boston Children’s Hospital and Boston University from 2015–2020 (IRB P00018377). Developmental and EEG data were collected longitudinally at 12, 18, 24, and 36 months. Data from one timepoint per child were drawn from the ISP2 dataset to create a cross-sectional cohort of typically developing children matched 1:1 on age and sex with the TSC cohort. Children selected for the typically developing comparison cohort met the following criteria: no developmental delay confirmed by parent report and/or scores on standardized developmental assessments (e.g., Vineland, Mullen Scales of Early Learning), no history of seizures, no first degree relative with autism spectrum disorder, birthweight >5.5 pounds, gestational age >31 weeks, and no genetic or neurological condition.

### Measures

#### Clinical

**Figure 1:**
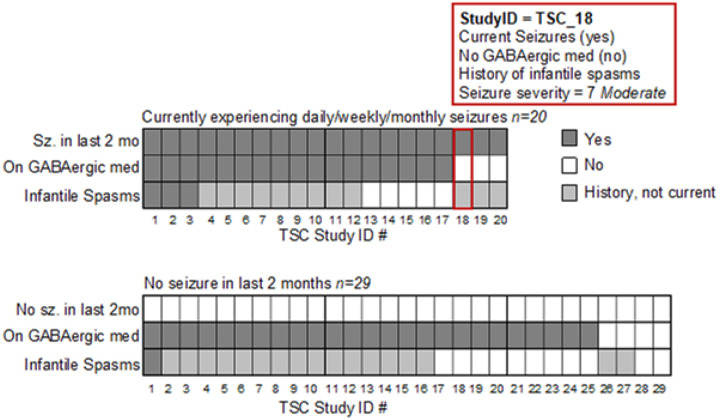
Seizure frequency, medication, and infantile spasms in individual TSC participants. Data reflect 49 TSC participants in a randomized control trial of the JASPER behavioral intervention. At baseline, participants presented with heterogenous profiles of seizure frequency, GABAergic medication use, and presence of infantile spasms. The profile of each participant is depicted as a column of three shaded rectangles reflecting the presence (dark gray), absence (white), or history (light gray) of each clinical feature. For example, participant #18 outlined in red was reported at baseline to experience seizures at least monthly over the last two months; not to take a GABAergic medication; and to have a history of infantile spasms but not currently experience infantile spasms. Seizure severity scores are derived from the E-Chess (21) and incorporate the frequency of seizures, types of seizures (including infantile spasms), and number of anti-epileptic mediations (all classes).

Seizure, infantile spasm, and medication data were collected from parent report at baseline (Fig 1). Since GABAergic medications are known to cause increased beta power (20), seizure medications were classified by mechanism of action into GABAergic medications and non-GABAergic medications (Table S1). As children with TSC have varying degrees of seizure activity, a composite seizure severity score was created using the E-Chess (21) to integrate frequency, medications, and types of seizures according to parent report. The seizure severity score ranged from 0 to 12 and reflected the sum of three variables: frequency of seizures in the last two months (0=no seizures, 4=more than daily seizures); number of current anti-epileptic medications (range 0–6); and total number of seizure types reported (1 point per type of seizure such as generalized, drop seizures, infantile spasms, etc.; range 0–5). Seizure severity scores were classified as low (0–2), moderate (3–7), or high (8–12).

The Mullen Scales of Early Learning (MSEL) was administered to assess developmental level. In order to avoid floor effects, Developmental Quotients (DQ) for each subscale were calculated (age equivalent / chronological age), then subscales were averaged to create a nonverbal DQ (visual reception + fine motor) and a verbal DQ (expressive language + receptive language). Autism diagnoses were not reliably available at the baseline timepoint given the age range of this cross-sectional cohort (35% of sample under 18 months).

#### EEG Recording

Resting state EEG data were collected continuously for at least 2 minutes at UCLA or Boston Children’s Hospital in a dimly light, electrically-shielded, sound-attenuated room. The child sat on a caregiver’s lap and watched a screensaver-style video of bubbles. EEG data were recorded using a 128-channel Hydrocel Geodesic Sensor Net (EGI, Inc., Eugene, OR, USA) that contains sponge-based carbon fiber electrodes. The sponges were first soaked in a solution of 6mL KCl/L of water and 5 mL of baby shampoo to facilitate conductance, then the net was placed over the child’s head, and electrodes were carefully seated on the scalp with impedances under 100 ohms. The net was connected to a DC-coupled amplifier (Net Amps 300 amplifier EGI) at a sampling rate of 500 samples per second, and referenced online to the vertex electrode (Cz). Similar data recording protocols and preprocessing pipelines were used for the matched typically developing cohort.

#### EEG Preprocessing

Raw EEG data were collected in NetStation (Magstim EGI) and exported to MATLAB (version 2021b) for processing using BEAPP version 4.1 (22) with embedded HAPPE software (23). This process involves first applying a 100 Hz low-pass filter, downsampling to 250 Hz, then using the HAPPE module of BEAPP to remove 60 Hz line noise and perform artifact detection and bad channel rejection using wavelet-enhanced Independent Component Analysis (ICA) and the Multiple Artifact Rejection Algorithm (MARA; (24)). To optimize ICA given the short length of the recording (2 minutes), only a subset of electrodes was included in this processing, in addition to 10–20 electrodes: 4, 19, 13, 112, 55, 67, 77, 28, 117, 47, 98, 75, 65, 90, 37, 87, 41, 103. Following ICA artifact detection and removal of bad channels, data were re-referenced using the average across channels. Mean detrending was used to process the continuous EEG, then data were segmented into 2-second segments. Following segmentation, using an artifact threshold of 40 uV (adjusted for HAPPE), segments were rejected based on joint probability and post-segmentation amplitude. Files were retained that met the following criteria: participant had 20 or more good segments, >80% good channels, mean or median retained artifact probability <0.3, percent independent components rejected as artifact <80%, and percent variance retained after artifact rejection >25%.

#### EEG Power Analysis and Parameterization

Using BEAPP software in MATLAB (22), power spectral densities for each electrode were estimated using multitaper spectral estimation with three orthogonal tapers. The power at each electrode was calculated for each frequency bin (0.5 Hz frequency resolution) for each two-second segment, then averaged across all segments for that electrode and frequency bin. Power values were normalized with a log10 transformation.

The total power spectrum can be decomposed into the periodic and aperiodic components by fitting an exponential decay curve (y=1/f) to the power spectrum to model the aperiodic component. The 1/f curve (aperiodic component) can be described with the offset value (similar to intercept) and exponent (reflects how steep or shallow the curve is, similar to slope). The modeled 1/f curve can then be subtracted from the total power spectrum, leaving only the periodic, oscillatory curve. For these data, the power spectral density was decomposed into periodic and aperiodic components using the SpecParam algorithm (also known as FOOOF v 1.0.0) from 2 to 55 Hz (25). Settings for the algorithm were as follows: peak width limits: 0.5–18.0; max number of peaks: 7; and peak threshold: 2.

#### Extraction of EEG features

After parameterization into aperiodic and periodic spectra, features of the EEG were extracted for analysis. Power was computed as an integral using three spectral density curves (periodic, aperiodic, and total power spectrum) for the following frequency ranges: theta (4 – 5Hz), low alpha (6 – 8 Hz), high alpha (9 – 11), alpha (6–11), low beta (12 – 19), high beta (20 – 29 Hz), broad beta (12 – 29 Hz), and gamma (30 – 44 Hz). Next, peaks were identified within the broad alpha and broad beta ranges by identifying, for each child, the local maximum within the designated frequency range. The power value and frequency value at that peak (log10(uV^A^2)/Hz) were recorded. Based on past literature, two regions of interest (Fig S1) were analyzed for all features: frontal (electrodes 24, 124, 11, 28, 117, 19, 4) and posterior (electrodes 70, 75, 83, 67, 77).

### Statistical Analyses

#### 1. Comparison of the power spectrum in TSC to typical development

To identify differences in the power spectral density between the TSC and TD groups across the frequency range (2–50Hz) for both full spectral power and periodic power, cluster permutation testing (n=1000 permutations) was implemented via the MNE software package in Python (v3.8.5) Jupyter Notebooks (v2.2.6).

To identify group differences in aperiodic components (frontal and posterior intercepts and slopes), logistic regressions were conducted to predict group status (TD vs TSC), implemented in RStudio (4.0.3).

#### 2. Seizures and medication use within the TSC cohort

The TSC sample was stratified by low (0–2), moderate (3–7), and high (8–12) seizure severity composites (integrating frequency, number of medications, and seizure type), and by use of GABAergic medications, which are known to affect spectral beta power. A two-way ANOVA was conducted to test the main effects and interaction of GABAergic medications and seizure severity on frontal beta peak. Post hoc comparisons were conducted and adjusted for multiple comparisons using Tukey’s HSD implemented in RStudio (4.0.3).


$$\text{frontal beta peak amplitude}=\beta_{0}+\beta_{1}\text{Seizure Severity}+\beta_{2}\text{GABA}+\beta_{3}\text{Seizure Severity}\times \text{GABA}+\varepsilon$$

## RESULTS

### 1. Children with TSC show greater beta power and greater aperiodic offset than typically developing controls

**Figure 2:**
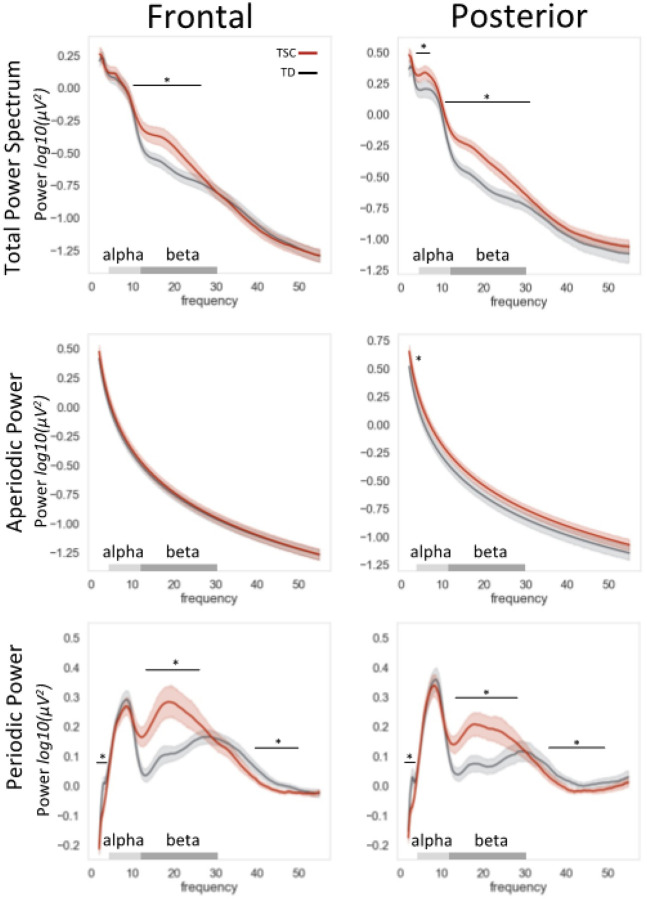
TSC and Typical Development parameterized power spectra. TSC and TD groups showed significantly different resting power in several frequency ranges, denoted by an asterisk and black bar spanning the significant frequency range. In aperiodic power (middle row), TSC and TD groups differed significantly in the posterior intercept, denoted by an asterisk.

**Figure 3:**
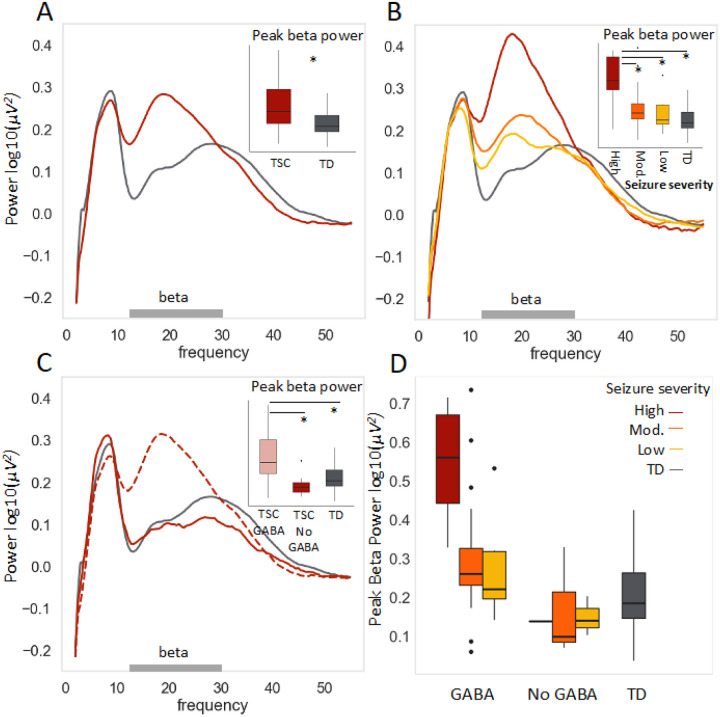
Parameterized frontal periodic power spectrum stratified by seizure composite and GABAergic medication use. (A) As a group, children with TSC (red, n=49) showed significantly greater peak beta power than age- and sex- matched typically developing controls (gray, n=49). (B) When the TSC participants were stratified by seizure severity composite, seizure severity appeared to drive the elevated peak beta power finding. The high seizure severity group (red, n=13) showed significantly greater peak beta power than the moderate (orange, n=23) and low (yellow, n=12) seizure severity groups, neither of which differed significantly from typically developing controls (n=49, gray). (C) When participants with TSC were stratified by GABAergic medication use, GABAergic medication use appeared to drive the elevated peak beta power finding. The participants with TSC on a GABAergic medication (red dashed line, pink box plot, n=42) showed significantly greater peak beta power than participants with TSC not on a GABAergic medication (n=7, solid red), who were not significantly different from typically developing controls (n=49, gray). (D) Among participants with TSC on GABAergic medication (left), those with high seizure severity (n=12, red) showed the greatest peak beta power. Seizure severity and GABAergic medication use were independently associated with elevated peak beta power; there was no significant interaction.

Cluster-based permutation testing revealed that, compared to age- and sex-matched typically developing controls, children with TSC showed significantly greater spectral and periodic power in the beta range in both frontal and posterior regions (Fig2, Fig3a, Table S2; spectral: frontal 10.6–25.1Hz and posterior 9.7–31.4Hz; periodic: frontal 10.6–26.1 and posterior 11.2–27.0Hz). In the alpha range, children with TSC showed significantly greater posterior power (spectral: 3.3–8.0Hz). In the theta range, children with TSC showed significantly diminished power (periodic: frontal 2.2–4.1Hz and posterior 2.1–3.6Hz). In the gamma range, the TSC group showed diminished power (periodic: frontal 34.5–49.7Hz and posterior 34.3–50.8Hz).

Based on these results, post-hoc analyses were conducted to compare beta power and peak beta frequency between groups. It was observed from individual waveforms (Fig S2) that peak beta frequency often occurred on the cusp between low beta (12–19 Hz) and high beta (20–29 Hz), around 20 Hz; thus, the decision was made to analyze peak beta frequency across the broad beta range (12–29 Hz). Both frontal and posterior peak broad beta frequency were lower for the TSC group than the TD group in paired t-tests (*t’s* > 3.8, *p*’s < 0.0005: TSC frontal M=20.9 Hz, posterior M=20.5; TD frontal M=24.6, posterior M=24.1). For comparison with other literature, we also tested high beta 20–29Hz and found significantly lower peak beta frequency for TSC than TD children (*t’s* > 5.8, *p*’s < 5E-6: TSC frontal M=23.7Hz, posterior M=23.9Hz; TD frontal M=26.7Hz, posterior M=27.1Hz). Of note, broad beta frequency showed trends of a positive correlation with Mullen NVDQ (frontal: r=0.30, p=0.05; posterior: r=0.27, p=0.07), and significant negative correlations with seizure severity (frontal: r=−0.34, p=0.02; posterior: r=−0.31, p=0.03).

#### Aperiodic features.

The four most informative aperiodic variables (intercepts and slopes for frontal and posterior regions) were entered into a logistic regression model predicting group status (TD, TSC). The odds ratio generated from the logistic regression can be interpreted as the odds that an individual falls in the TSC group, rather than the TD group, given a 1-point change in modeled aperiodic intercept (i.e., from 0.01 to 1.01), which has a range in this sample of −0.04 to 1.00. In the model, posterior intercept emerged as a strong and sole significant predictor of group status (Fig 2), such that when holding frontal intercept, frontal slope, and posterior slope constant, a 1.0 point change in posterior aperiodic intercept corresponded to a 222.9 increase in odds of belonging to the TSC group (OR = 222.9, p = 0.0037, 95% CI [7.2, 11,312.5]).

### 2. Higher peak beta power is associated with both higher seizure severity, and GABA agonist use

Next, we examined the main effect of seizures on the power spectrum (Fig 3a, 3b) for participants with TSC with available seizure severity data (n=48 of 49). Participants with high seizure composite scores showed a marked increase in frontal peak beta power (n=13; M(SD)= 0.52(0.17)) compared to those with moderate scores (n=23; (M(SD)= 0.28(0.16)), low scores (n=12; M(SD)= 0.24(0.12)), or typically developing controls (M(SD)= 0.20(0.09)). There was a significant main effect of seizure severity on frontal broad beta peak amplitude F(3, 90) = 29.28, p < 0.001, and post-hoc Tukey HSD tests showed a significant difference between all groups and the high seizure severity group (all adjusted p’s < 0.0005).

Since GABAergic medications are often used to manage epilepsy, we stratified the TSC sample by GABAergic medication use (Fig 3c). Medication data were available for all participants, and only 7 of 49 TSC participants were not on a GABAergic medication. Despite the small sample, we observed a significant increase in beta amplitude in participants on GABAergic medications (n=42; M(SD)= 0.38(0.19)) versus off (n=7; M(SD)=0.15(0.09)); individuals off GABAergic medication showed a beta peak more similar to their TD matches (M(SD)=0.20(0.09)) than to other individuals with TSC. Specifically, there was a significant main effect of GABA agonist medication on frontal broad beta peak amplitude *F*(2, 90) = 16.42, *p*< 0.001), and post-hoc Tukey HSD tests showed a significant difference between all groups and the GABA agonist group (all adjusted *p*’s < 0.00005).

Finally, individuals with high seizure scores on GABAergic medications (n=12) showed the most elevated beta peak (Fig 3d). As GABA agonists are prescribed for elevated seizure activity, it was important to determine whether GABA and seizure activity were independently associated with increased beta power. Both GABA status and seizure severity were significantly, independently associated with peak beta amplitude when included in the same model, and the interaction was not significant (*F*(2, 90) = 2.20, *p* = 0.12).

Though not a primary aim of this study, we explored associations between Mullen NVDQ scores and peak alpha frequency within to replicate associations reported by Dickinson et al. (2019). No significant association was observed between NVDQ and peak alpha frequency (frontal r=−0.21 p=0.17; posterior r=−0.11 p=0.48, uncorrected p values). Further research with larger samples with a narrower age range may clarify the association between peak alpha frequency and cognition in TSC.

## DISCUSSION

In resting state EEG data collected from a cohort of children with TSC aged 12–37 months and age and sex-matched typically developing children, the TSC group showed markedly increased beta power that appeared driven by individuals with high seizure activity, as well as those on GABAergic antiepileptic medication, as hypothesized. Seizure activity and GABAergic antiepileptic medication use are confounded by indication, so it is notable that there was no significant interaction between GABAergic medication use and seizure severity; in other words, seizure severity and GABAergic medication are both independently associated with increased beta power in the TSC group.

### Characterization of TSC resting EEG power spectrum

TSC participants showed increased periodic beta and gamma power, reduced theta periodic power, increased absolute alpha power, and increased posterior aperiodic intercept compared to matched typically developing controls. Our TSC characterization fits with previous results and provides additional insights. We observed differences in periodic theta (a notable peak in the TD cohort, and absence of a peak in the TSC cohort), which has not been previously reported. In the alpha frequency, we observed increased alpha power only when measured as absolute power in the posterior region in our sample of 49 children with TSC and 49 matched controls. In contrast, Dickinson et al. (2019) found no significant group differences in relative alpha power at 12, 24, or 36 months in a smaller longitudinal sample (n=23 children with TSC and n=20 controls). Dickinson et al. (2019) also identified a trending positive association between TSC 24-month peak alpha frequency and 36-month NVDQ verbal and nonverbal that only survived correction for multiple comparison in the posterior region. In contrast, in our cross-sectional sample, no significant association was observed between NVDQ and peak alpha frequency. Further research with larger samples with a narrower age range may clarify the association between peak alpha frequency and cognition in TSC.

### Beta frequency, seizures, and GABA agonist use

In perhaps our most robust finding, TSC participants showed a notable elevated beta peak in both spectral absolute power and periodic power after decomposition with SpecParam. The beta peak was observed consistently in both frontal and posterior regions around 20Hz in broad beta (12–29Hz) and around 24Hz in high beta (20–29Hz, excluding low beta peaks from the average), whereas typically developing controls showed a peak beta frequency closer to 24 Hz in broad beta and 27 Hz in high beta. Increased beta peak power was associated with both increased seizure severity and GABA agonist use in the TSC cohort. In both humans and animal models, GABA agonists have consistently been associated with increased beta power (26–29). In pharmacological studies of healthy adults, the administration of GABA agonists appears to increase beta power consistently (30–32), but results differ on whether peak beta frequency is unaltered (30,31) or decreased (33), with the spectral peak widened (33). The direct positive relationship between increased GABA concentration and increased peak beta frequency has been reported using magnetic resonance spectroscopy and magnetoencephalography(34). Thus, it is likely that GABAergic medication use in our sample could be driving the increased beta power and decreased peak beta frequency, at least in part.

However, in the current sample, GABA agonists alone likely do not explain the increased beta power and lower peak beta frequency observed in the TSC cohort. Surprisingly, seizure severity – likely reflecting excessive excitatory signaling – also correlated positively with increased beta power. Recent reports of typical development suggest that beta power may reflect the maturation of GABAergic interneuron networks (Wilkinson et al., 2023); thus, the increased power observed in individuals with TSC relative to typically developing peers, and most notably in individuals with high seizure severity, may reflect delayed development. This hypothesis was supported by a post-hoc negative correlation of −0.32 (uncorrected p=0.03) between the Mullen NVDQ and frontal broad beta power.

Further, it remains possible that TSC itself is characterized by increased beta power and lower peak beta frequency. For comparison, Duplication 15q carries an EEG signature of increased spontaneous beta oscillations that result in increased power, and peak high beta frequency of 23 Hz in a sample with a broad age range (9 months to 14.5 years) (31). Notably, epilepsy shows the opposite association in Dup15q compared to our TSC cohort, with epilepsy diagnosis predicting less high beta power in Dup15q (35), and greater seizure severity predicting more beta power in TSC. In Fragile X, an unusually high peak beta frequency of 30 Hz was observed in a cohort of 3–7 year olds (36), and the peak beta frequency decreased with age suggesting that peak beta frequency may be an index of brain maturity (37). In a large developmental normative sample of nearly 600 children described in Wilkinson et al. (2023), the high beta peak we observe in TSC could be interpreted as a sign of delayed maturation since Wilkinson et al. report nonlinear early developmental changes in the high beta peak with both peak frequency and amplitude increasing during the first year of life, peaking at 12 months, and then decreasing substantially over the following year.

### Resting EEG as a biomarker in the context of seizures and medication use

At the start of the RCT, we hypothesized that resting EEG spectral power might serve as a biomarker - either as an outcome measure, or to predict treatment response to the behavioral intervention JASPER. These prospects could be undermined by the observed increase in beta oscillatory activity associated with high seizure activity, and most importantly, the use of medication known to impact beta power (i.e., some antiepileptics, some anxiolytics). It is neither feasible nor ethical to control this confound by requiring participants with epilepsy to stop medication use during a behavioral intervention trial such as our JASPER RCT, as medications often provide critical control of seizures. This challenge is true for not only TSC, but also all neurodevelopmental conditions with epilepsy. This challenge also extends to conditions with co-occurring anxiety for which benzodiazepines – GABA agonists known to increase beta power – are prescribed. However, neural markers of intervention response are desperately needed, and EEG features offer great promise as EEG is tolerated more readily than MRI. We recommend that future studies of resting EEG in neurodevelopmental conditions of all ages collect comprehensive seizure and GABAergic medication data at each EEG data collection point, including changes in medications, so that analyses can account for exogenous influences on candidate biomarkers.

Limitations of this analysis include its cross-sectional design across a broad age range (12–37 months), absence of tuber location MRI data, and reliance on parent-report for medication, seizure, and TSC diagnosis.

## CONCLUSIONS

This paper adds to the current literature on resting EEG spectral power biomarkers in neurodevelopmental disorders by identifying common confounding variables that impact beta power (seizures, antiepileptic GABAergic medication use). To the TSC literature specifically, this paper contributes a characterization of resting EEG in toddlers, benchmarked against typical development. Through this careful comparison, we were able to identify the elevated beta power in children with TSC, and relate it to sample characteristics (medication use, increased seizure severity). Future directions include investigation of identified resting EEG features in relation to RCT behavioral outcomes, and collection of larger samples of resting EEG in individuals with TSC to characterize beak power and peak beta in subgroups of medication use and seizure severity.

## Figures and Tables

**Table 1:** Participant characteristics

	Tuberous Sclerosis Complex participants	Typically Developing Controls
N	49	49
Age, months, mean(SD)	22.2 (7.4)	23.0 (8.1)
Sex, % male	51.0%	51.0%
Mullen Verbal Developmental Quotient, mean(SD)	63.2 (23.6)	117.9 (17.8)
Mullen Nonverbal Developmental Quotient, mean(SD)	73.1 (22.1)	114.8 (14.2)
**Seizures**		
% current seizures (last 2 months)	40.8%	
% on GABA antagonist	85.7%	
% infantile spasms: current past	8.2 59.2 32.7%	
no history of spasms		
Number of seizure medications	2.4 (1.4)	
Surgery	8.2%	

## Abbreviations

BEAPP: Batch Electroencephalography Automated Processing Platform
DQ: Developmental Quotient
Dup15q: Duplication 15q
EEG: Electroencephalography
ICA: Independent Component Analysis
ISP2: Infant Screening Project 2
JASPER: Joint Attention, Symbolic Play, Engagement & Regulation
JETS: JASPER Early Intervention for Tuberous Sclerosis Complex Study
mTOR: Mammalian Target of Rapamycin
MSEL: Mullen Scales of Early Learning
MARA: Multiple Artifact Rejection Algorithm
NVDQ: Nonverbal Developmental Quotient
RCT: Randomized Controlled Trial
SD: Standard Deviation
TAND: TSC-Associated Neuropsychiatric Disorders
TD: Typically Developing
TSC: Tuberous Sclerosis Complex
